# General Medicine and Therapeutics

**Published:** 1893-08

**Authors:** 


### GENERAL MEDICINE AND THERAPEUTICS.

EDITED BY DR. LUTHER B. GRANDY.

### THE PRACTICE OF HYPNOTISM.

Ernest Hart (*British Medical Journal*) says that, since no evident advantage has during forty years of extensive, patient and elaborate trial and research been obtainable by the hundreds of physicians and physiologists who have devoted themselves to the study of the question, it is justifiable to say that the practice of hypnotism, mesmerism, electro-biology and so-called animal magnetism, being almost invariably useless and often dangerous, even in the hands of the most highly skilled, careful and conscientious physicians, is a practice which ought to be forbidden to the unqualified, and is a most unfit and improper subject for platform shows. It is equally unfit for private amusements, and dangerous and improper as a society game. It is liable to gross abuse, and is frequently the means of fraud and imposture, extortion of money and degradation of body and mind. It is apt to induce serious injuries to both. The confirmed and trained hypnotic subject is a maimed individual in mind and body, and likely at any time to be dangerous to himself and to society.—*American Lancet.*

### TO STIMULATE APPETITE.

In a lecture on "Concentrated Food in the Treatment of Pulmonary Consumption" (*Pitts. Med. Rev.*), Dr. Thomas J. Mays says that much can be done to stimulate the appetite. For this purpose he often gives the following:

℞. Acid phosphoric dil., ½ ounce.

Acid nitro-muriatic dil., ½ ounce.

Acid sulphuric aromatic, ½ ounce.

Tinct. ferri chloridi, ½ ounce.

M. Sig.: Thirty drops in a half glass of cold, sweetened water during meals.

---

### THE VALUE OF QUININE IN MALARIAL HÆMATURIA.

Prof. H. A. Hare, in a very scholarly paper, concludes that in the milder forms of the disease quinine should not be employed during the hæmaturia, nor in those cases in which idiosyncrasies exist. In cases which are malignant in their manifestations in other lines than the mere hæmaturia, and depend upon a malarial paroxysm superimposed upon a malarial dyscrasia, then quinine is needed; but in the hæmaturia following prolonged subacute or chronic malarial poisoning, it is not to be given in full doses, but only as a tonic, the physician relying upon change of climate, nutritive foods, tonics, and other drugs to renovate the patient's tissues.—*Therapeutic Gazette, American Journal Medical Sciences.*

---

### HYDROGEN PEROXIDE IN CHOLERA.

Two papers have recently appeared describing the usefulness of the dioxide of hydrogen in the treatment of cholera; one by Dr. Elmer Lee, of Chicago (*Chicago Chemical Review*), and the other by Dr. Cyrus Edson, of New York (*Doctor of Hygiene*). Dr. Lee says that in his experiment in Russia and Hamburg in 1892, the medicinal peroxide of hydrogen in cupful doses of the 4 per cent. solution every two hours proved a better antiseptic than any other drug. Patient was urged to drink freely of hot water. Bowels were also irrigated twice a day for the first and second day. Feeding and nursing as in any prostrating disease.

Dr. Edson says: I desire to emphasize the fact that we have in hydrogen dioxide a powerful antiseptic agent which may be administered without harm to the human system, and by means of which the alimentary canal can be more thoroughly disinfected than by any other agent in our present range of therapeutics. In other words, there is no other antiseptic that will effect the amount of germ destruction in the alimentary canal without inflicting injury.

---

### CAPITAL PUNISHMENT AND CASTRATION.

I wish to place myself on record as opposed to capital punishment, legal or illegal. From a utilitarian standpoint I have failed to see wherein capital punishment has, in the aggregate, repressed those crimes for which it is prescribed.

To my mind, there is only one logical method of dealing with capital crimes and criminals of the habitual class—namely, *castration.* This method of punishment leaves behind it evidences which will prove wholesome warning to criminals of like propensities. It prevents the criminal from perpetuating his kind. The murderer is likely to lose much of his savageness; the violator loses not only the desire, but the capacity for a repetition of his crime, if the operation be supplemented by penile mutilation according to the Oriental method. A few emasculated negroes scattered around through the thickly-settled negro communities of the South would really prove the conservation of energy, as far as the repression of sexual crimes is concerned. Executed, they would be forgotten; castrated and free, they would be a constant warning and ever-present admonition to others of their race. Thus, I believe that castration would be peculiarly applicable to the class of criminals under consideration; while, at the same time, claiming that it is the rational method of dealing with the crime question in many of its phases.—*Dr. Lydston, in Virginia Medical Monthly.*

